# Pneumatocele Manifestation in Early Recovery Period of COVID-19: A Case Study

**DOI:** 10.7759/cureus.41259

**Published:** 2023-07-01

**Authors:** Jennaire Lewars

**Affiliations:** 1 Internal Medicine, Carle Foundation Hospital, Urbana, USA; 2 Graduate Medical Education, Saint James School of Medicine, Chicago, USA

**Keywords:** sars cov2, viral illness, lesions of the lung parenchyma, pneumatocele, covid-19

## Abstract

In this case report, we review a case of pneumatocele in a 54-year-old Caucasian male patient diagnosed with infection of COVID-19 29 days prior, presenting with complaints of dyspnea and sharp pleuritic chest pain located in the left mid-axillary region exacerbated by deep inspiration with an episode of significant forceful cough a day prior. Preliminary labs were unrevealing for leukocytosis or neutrophilia, with normal troponins, and COVID-19 negative upon presentation. Radiographic imaging was significant for bilateral infiltrates in the left upper lobe, air fluid levels with initial concern for abscess but with subsequent inference of pneumatocele. Imaging was negative for pneumothorax. The patient was monitored, remained stable throughout admission, and discharged after work-up for fungal and bacterial infection were found to be negative with expectation of self-resolution of the pneumatocele. In this study, we overview the pulmonary impact of COVID-19 in the scope of pneumatocele occurrence in the early recovery phase of the viral illness.

## Introduction

The COVID-19 pandemic has become a primary concern in modern day history and has induced a paradigm shift in the scope of medicine with its variable presentations, impact, treatment modalities, preventative measures and long-term sequelae. COVID-19 has spread worldwide with over 312 million cases, 5.5 million deaths, and upwards of 2 million new cases in a single day as of January 2022 [1]. COVID-19 is a clinical syndrome caused by the virus Severe Acute Respiratory Syndrome Coronavirus 2 (SARS-CoV2). The implications and complete scope of COVID-19 are still evolving. COVID-19 has been associated with inflammation of the lungs, atelectasis, pleurisy, cough and numerous other pathologies. It is associated with targeted damage within the epithelial cells of the alveoli, impacting structural integrity of the lung parenchyma and rendering the alveoli susceptible to rupture. A pneumatocele is a thin-walled, cystic, air-filled lesion present within the lung parenchyma. Its occurrence may present secondary to multiple aetiologies such as chest trauma, bacterial pneumonia, chemical toxicity and positive-pressure ventilation [2]. These lesions are typically asymptomatic and usually self-resolve without requirement of specific treatment or intervention [2].

## Case presentation

**Figure 1:**
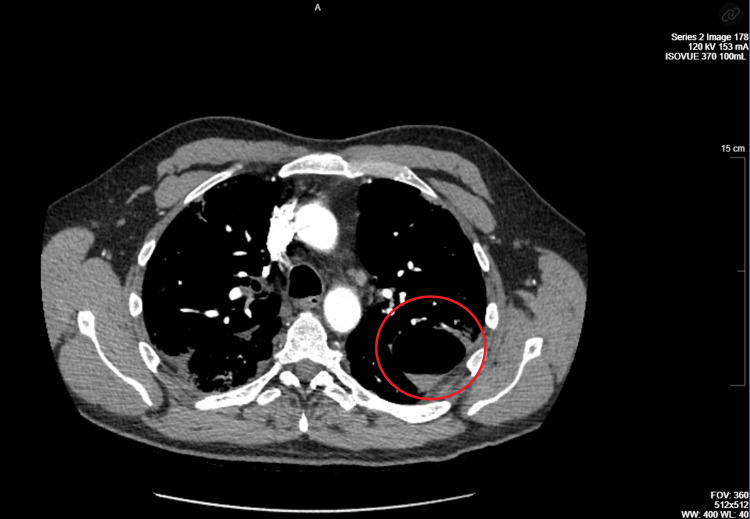
CT angiography of the chest with contrast. The red circle points to pneumatocele within the left lung.

**Figure 2:**
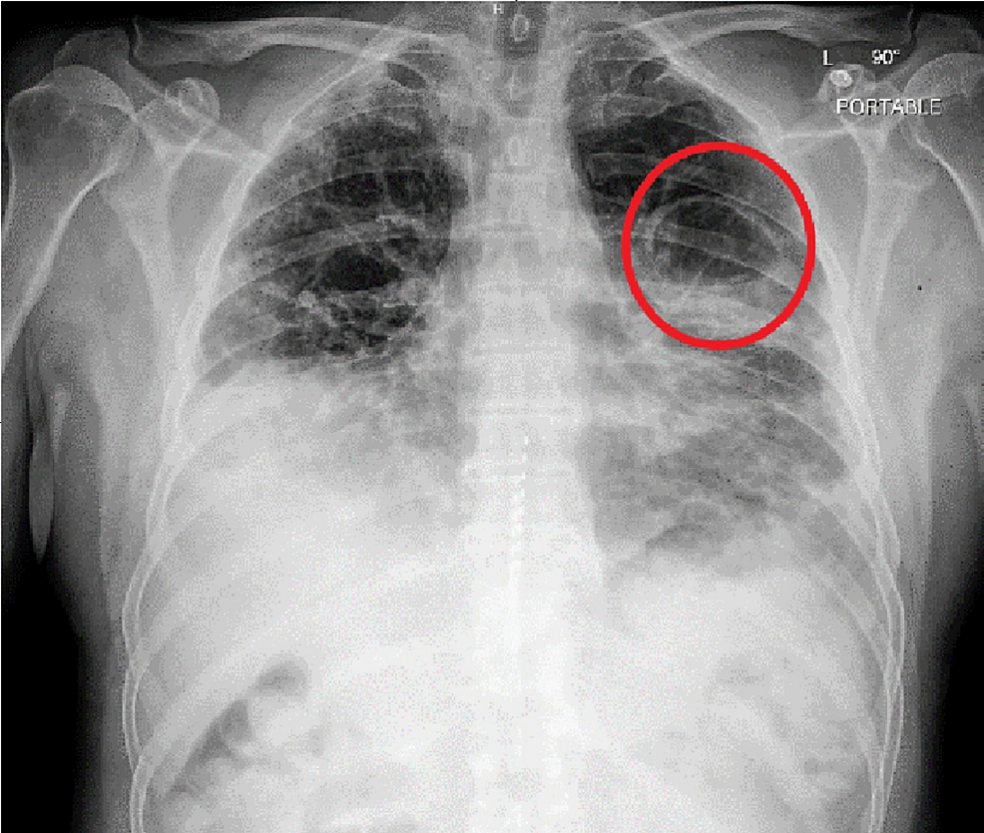
Chest X-ray of pneumatocele in left upper lobe of lung (red circle)

The patient was a 54-year-old Caucasian male with a recent diagnosis of COVID-19 with acute hypoxic respiratory failure, complicated by acute pulmonary embolism and deep venous thrombosis 29 days prior to his hospital presentation, managed by 10 days of dexamethasone 6 mg and three days of remdesivir. He also had a history of obstructive sleep apnea not utilizing continuous positive airway pressure (CPAP), in absence of obesity hypoventilation syndrome. He had a BMI of 27 and was a non-smoker. He presented to the emergency department with acute complaint of dyspnea, sharp pleuritic chest pain exacerbated by deep inspiration, localized to the left mid-axillary region, commencing a day prior to his presentation. He reports episode of significant coughing on the day before the manifestation of his pleurisy along with rusty colored sputum similar to the productive content present during his initial bout of COVID-19. Upon admission, he was afebrile, had an oxygen saturation 95% on room air, with a pulse approximately 80 beats per minute, and blood pressure stable at 120s/80s mmHg. His preliminary laboratory workup with a complete blood count and comprehensive metabolic profile was unrevealing for leukocytosis or neutrophilia. His troponin was unremarkable (<0.01 ng/mL). Physical findings were revealing for fine crackles appreciated bilaterally, particularly on the right mid-lung and the bases, and diffuse in distribution throughout the left lung, with intermittent coughing eliciting pleuritic chest pain. A CT angiography for pulmonary embolism (CT CTA PE) (Figure 1) was undertaken which was negative for pulmonary embolism, but was significant for bilateral infiltrates, consistent with COVID-19 and an upper lobe cavitary lesion with air-fluid levels, initially considered to be an abscess, and additionally with the presence of right lower lobe consolidation. Chest X-ray (Figure 2) was performed subsequently, which was revealing for persistent coarse bilateral pulmonary infiltrates with possible slight worsening of the left lung base, as well as a persistent pulmonary cystic lesion in the left upper lobe.

There was an initial concern for abscess per imaging and radiographic impression, with additional suspicion for the presence of a pneumatocele as a top differential. Blood cultures were drawn, with the patient placed on empiric Vancomycin 1,250 mg/270 mL every 12 hours with Piperacillin-Tazobactam 4.5 g/100 mL every 8 hours. The patient had received Ampicillin-Sulbactam 3 g and hydrocodone-acetaminophen at an outside facility before his presentation to this location. He was found to be negative for COVID-19 via polymerase chain reaction (PCR) test upon admission. Legionella and strep pneumonia urine antigen were negative. Tuberculosis quantiferon, histoplasma, blastomyces quant antigen, aspergillus and fungal workup were also unremarkable. HIV screening was done and was also negative. Interventional radiology evaluated the patient and reviewed the aforementioned imaging during the admission who deemed a high likelihood of pneumatocele being reflected on the imaging instead of an abscess, along with no overt signs or symptoms of infection. Due to the self-resolving nature of pneumatoceles, absence of pneumothorax, vital stability, and absence of an infectious process, the decision was made to pursue conservative management of the condition. The patient was discharged after a three-day admission with suggestion of over-the-counter analgesics and recommendation to return to the emergency department if any development of worsening symptoms such as but not limited to increased shortness of breath or work of breathing, chest pain and lightheadedness. Upon follow-up one week post discharge, the patient reported that his symptoms had improved. 

## Discussion

A pneumatocele is a thin-walled, cystic, air-filled lesion present within the lung parenchyma. Its occurrence may present secondary to multiple aetiologies such as chest trauma, bacterial pneumonia, particularly, Streptococcus and Staphylococcus aureus pneumonia, chemical toxicity, such as hydrocarbon ingestion and positive-pressure ventilation [2]. Pneumatoceles are typically rare in the adult population; they tend to occur in infants, children, and adolescents, and there are only a few adult cases noted in literature. In the context of COVID-19, cystic lung changes occur in up to 10% of cases [3]. The incidence and pathological mechanism of this occurrence are unclear with regards to COVID-19, but it is postulated that diffuse alveolar damage secondary to the infection impacts structural integrity of the lung parenchyma resulting in necrosis within the walls of the airways and induces pneumatocele formation [4]. With the symptomology of COVID-19 inclusive of complaints, such as pronounced, forceful recurrent bouts of coughing, coupled with this deficiency in the structural durability of the lung tissue due to COVID-19 within the early recovery phase, the intrathoracic pressure induced by coughing further reinforces the predilection to the formation of pneumatoceles. Imaging modalities that are performed to identify and delineate pneumatoceles from other pathologies such as abscesses, include CT scans of the chest. Chest X-rays may reveal findings of a thin-walled cyst, which may appear up to five-six days after an infectious etiology. Pneumatoceles generally tend to result in a good prognosis as they are usually uncomplicated, though there are risks for pneumothoraces, or tension pneumatoceles that can induce cardiac or respiratory compromise [2]. Usually, the management of uncomplicated pneumatoceles includes monitoring and treatment of the underlying causes such as pneumonia. In rare instances, tension pneumatoceles may require a percutaneous drainage [5]. In our case, the management as such was conservative, with analgesics for discomfort and monitoring of symptoms concerning pneumothorax, with expectation of self-resorption after resolution of suspected pulmonic inflammation. Further suggestions include cough suppressants to reduce the likelihood of further exacerbation of pneumatocele formation.

## Conclusions

The mechanism of manifestation of pneumatoceles in the early recovery phase of COVID-19 is still unclear though there are multiple postulated assertions. It is evident that there are increasing reports of the occurrence of pneumatoceles in its association with the viral illness due to SARS-CoV2 and is one of concern due to the potential sequelae of dissection and pneumothorax, which could result in a patient’s demise. It is important to understand the pathophysiology of this occurrence as we continue to manage patients with COVID-19.
